# Immobilization of Eversa^®^ Transform via CLEA Technology Converts It in a Suitable Biocatalyst for Biolubricant Production Using Waste Cooking Oil

**DOI:** 10.3390/molecules26010193

**Published:** 2021-01-02

**Authors:** José Renato Guimarães, Letícia Passos Miranda, Roberto Fernandez-Lafuente, Paulo Waldir Tardioli

**Affiliations:** 1 Postgraduate Program in Chemical Engineering (PPGEQ), Department of Chemical Engineering, Federal University of São Carlos (DEQ/UFSCar), Rod. Washington Luís, km 235, 13565-905 São Carlos, SP, Brazil; renatoge74@gmail.com (J.R.G.); lettypassos@gmail.com (L.P.M.); 2 Departamento de Biocatálisis, ICP-CSIC, Campus UAM-CSIC, 28049 Madrid, Spain; 3 Center of Excellence in Bionanoscience Research, External Scientific Advisory Academic, King Abdulaziz University, 21589 Jeddah, Saudi Arabia

**Keywords:** synthetic biolubricants, transesterification, waste cooking oil, Eversa, magnetic CLEAs, lipase features tuning via immobilization

## Abstract

The performance of the previously optimized magnetic cross-linked enzyme aggregate of Eversa (Eversa-mCLEA) in the enzymatic synthesis of biolubricants by transesterification of waste cooking oil (WCO) with different alcohols has been evaluated. Eversa-mCLEA showed good activities using these alcohols, reaching a transesterification activity with isoamyl alcohol around 10-fold higher than with methanol. Yields of isoamyl fatty acid ester synthesis were similar using WCO or refined oil, confirming that this biocatalyst could be utilized to transform this residue into a valuable product. The effects of WCO/isoamyl alcohol molar ratio and enzyme load on the synthesis of biolubricant were also investigated. A maximum yield of around 90 wt.% was reached after 72 h of reaction using an enzyme load of 12 esterification units/g oil and a WCO/alcohol molar ratio of 1:6 in a solvent-free system. At the same conditions, the liquid Eversa yielded a maximum ester yield of only 34%. This study demonstrated the great changes in the enzyme properties that can be derived from a proper immobilization system. Moreover, it also shows the potential of WCO as a feedstock for the production of isoamyl fatty acid esters, which are potential candidates as biolubricants.

## 1. Introduction

Current concerns about global warming and the reduction of petroleum reserves have driven the search for chemicals that can be biodegradable and environmentally friendly. For example, vegetable oils are promising substitutes to mineral-oil based lubricants after their conversion to fatty acid alkyl esters using long-chain alcohols (C8 to C14), such as octanol [1,2], branched alcohols, such as isoamyl alcohol [3,4,5] and 2-ethyl-1 hexanol [6], and polyols [7], such as neopentyl glycol [8], pentaerythritol [9,10,11] and trimethylolpropane [12,13]. Fatty acid alkyl esters having 22 to 26 carbon atoms can serve as biolubricant components [1] due to their suitable physicochemical properties (some of them after a proper chemical modification of the esters, for example, an epoxidation of C=C double bonds of the unsaturated fatty acid esters [14]), such as high viscosity index, high lubricity, high flash point, low volatility, good anti-wear performance, high thermo-oxidative stability, and good performance at low temperatures [1,5,14,15].

Synthetic biolubricants are obtained by direct esterification of fatty acids [16,17], transesterification between monoalkyl esters of the target fatty acid or glycerides and the desired alcohol [18,19], or hydroesterification, a sequential process of hydrolysis of oils/fats followed by esterification of the purified fatty acids [1,8]. These reactions have been performed using either homogeneous or heterogeneous catalysts, however, from an industrial point of view, heterogeneous catalysts are preferred due to the easier recovery of the catalyst and lower generation of chemical wastes [20]. The use of immobilized lipases (triacylglycerol acylhydrolases, E.C. 3.1.1.3) as heterogeneous catalysts is a promising alternative to the synthesis of esters due to the favorable characteristics for the enzymatic process, such as low energy demand, the possibility of using feedstock without previous purification (e.g., waste cooking oil containing a high percentage of free fatty acid), and lower complexity of the recovery and purification of the product [20,21,22].

Eversa^®^ Transform 2.0 was initially launched to be used as a liquid biocatalyst in the production of biodiesel [23,24]. However, it has been shown that if the enzyme is properly immobilized, the enzyme performance could be improved in diverse applications, such as biodiesel [25,26,27], free fatty acid (FFA) [28], or glyceride [29] production, among others process [24]. Eversa is a genetically modified industrial evolution of the lipase from *Thermomyces lanuginosus* (TLL) expressed in *Aspergillus oryzae* by Novozymes A/S [24,30]. Although both enzymes are similar in terms of primary sequence, a recent comparison between them showed that the functional properties could be fairly different [31]. Eversa has shown improved thermal stability under different conditions and lower sensibility to the presence of phosphate anions [31]. However, immobilized Eversa and TLL were similar in the production of biodiesel [31]. In addition, TLL has been successfully applied in the production of synthetic biolubricants. Similar conversions (about 90%) of synthetic biolubricants were obtained using TLL immobilized on different solid supports, such as citric acid-modified magnetite nanoparticles [8], Duolite A568 [17], and mesoporous hydrophobic poly-methacrylate [3,16].

Enzyme immobilization may become a powerful tool to greatly improve their performance, enzyme stability, activity, specificity, or selectivity [32,33]. Immobilization may also increase the range of conditions where the enzyme may be utilized [34], reduce inhibitions [35] or inactivation caused by the substrate [36], or be coupled to enzyme purification [37]. TLL and Eversa have been in some instances examples of this tailoring of enzyme features via immobilization [38]. In extreme cases, an enzyme that apparently is unsuitable for a process may suffer sufficiently large changes after immobilization so that it becomes useful as a catalyst for that specific process [39].

Immobilization of Eversa on solid supports [25,27,40,41,42,43] has been successfully developed previously. However, the cost of supports can increase the price of the final biocatalyst. Thus, the immobilization of Eversa by the CLEA technique appears as an alternative that presents some advantages such as producing a biocatalyst with high volumetric activity, does not require highly pure enzyme solutions, and allows the co-immobilization of different enzymes [44,45,46,47,48]. However, it has some problems, such as mechanical fragility and high diffusion limitations, which have been overcome by the use of pore-forming agents, polymers, and protein co-feeders during the stages of aggregation and cross-linking [46,49,50]. Another approach that has been reported to improve the mechanical resistance of CLEAs, as well as facilitate their separation and reuse is the co-aggregation of the enzyme with magnetic nanoparticles [46]. Magnetic CLEAs of several enzymes have been reported [46,50,51,52,53,54,55,56,57]. Specifically, CLEAs of Eversa containing magnetic nanoparticles (Eversa-mCLEA) showed good performance and reusability in the production of biodiesel [26].

In this study, liquid Eversa and Eversa-mCLEA were utilized as biocatalysts for the production of biolubricants for the first time. The transesterification of WCO with different alcohols useful to produce biolubricants was evaluated here. WCO production is quite high and it is a residue with negative ecological impact [58]. This way, the current paper presents a project with a triple ecological impact: the production of a biodegradable biolubricant using a green route and a feedstock that is currently used just as disposal with negative environmental impact [59,60]. The transesterification of this raw residue is feasible by the enzymatic route while it is complex by the standard chemical route due to the risks of saponification [61,62]. The activities of both Eversa formulations were evaluated using different alcohols as substrates. Lastly, the product was characterized in order to confirm the synthesis of isoamyl esters.

## 2. Results and Discussion

### 2.1. Physicochemical Characterization of the Utilized Oils

The physicochemical analyses of the feedstocks used for the transesterification reaction are shown in Table 1. The saponification index for refined soybean oil and WCO showed similar values, which are within the range (189–195 mg KOH/g) reported for soybean oil [63]. The WCO showed an acidity index around 33 times higher than the refined oil; however, the water content for both oils showed no significant differences. These results suggest that the cooking oil suffered a significant degree of deterioration during the frying process.

### 2.2. Performance of Liquid and Immobilized Eversa

Table 2 shows the comparison of the performance of liquid and immobilized Eversa in esterification reactions. Eversa-mCLEA provided ester yields 1.5 and 1.4 times higher than those reached with liquid Eversa, when octanol and isoamyl alcohols were used as acyl acceptors, respectively. It is worth mentioning that the experimental conditions were not optimized for each formulation; the same conditions were adopted only for comparison purposes. These results suggest the feasibility of using Eversa-mCLEA as a biocatalyst in the production of esters with lubricant properties. Differences in the enzyme microenvironment could explain the better performance of the immobilized enzyme in this reaction because the nanoparticles functionalized with octyl groups could favor a fast mass transfer of hydrophobic substrates from the bulk to the active site of the enzyme. Thus, only Eversa-mCLEA was used for further experiments.

### 2.3. Effect of the Alcohol Chain on the Transesterification Activity of Immobilized Eversa

**Figure 1:**
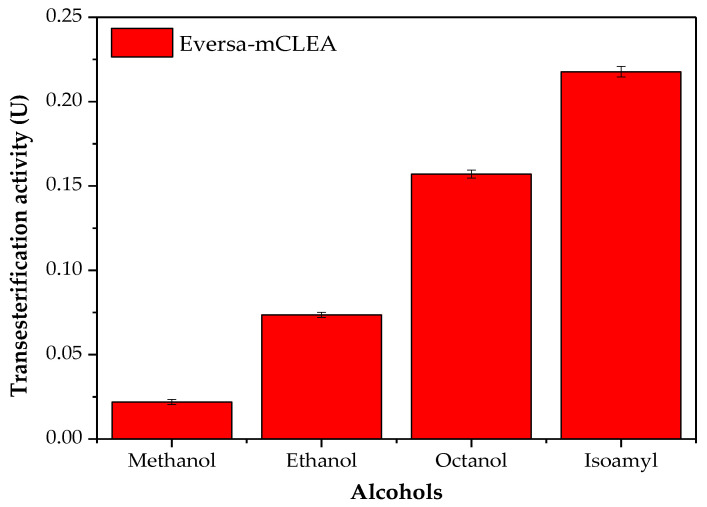
Transesterification activity of Eversa-mCLEA in the presence of WCO and different alcohols. Assay conditions: enzyme load of 2.13 U_est_/g oil, oil/alcohol molar ratio of 1:6, temperature of 40 °C, agitation of 250 rpm for 2 h. Samples were collected during the reaction to quantify the esters.

The influence of the length and branching of some alcohols on the transesterification activity of Eversa-mCLEA was evaluated (Figure 1). In absence of branching in the alcohols, the higher the length of the chain was, the higher the transesterification activity was observed, as expected according to the previous findings reported in the literature, in which using other lipases, an increase in the yield of esters would be expected with the increase in the carbon chain and absence of branching in the alcohol [64,65,66]. Eversa-mCLEA was less active using short-chain primary alcohols (methanol and ethanol), which are the alcohols usually utilized in biodiesel production [67,68,69]. This lower activity can be explained by the fact of methanol and ethanol change the hydration layer of the enzyme [21,70,71,72], justifying the common practice of adding the alcohol in steps, mainly when methanol is used as an acyl acceptor ([24]). Similar results were obtained by Trivedi et al. [66] using the formulation preceding Eversa (soluble lipase Callera Trans L, EP 258068) in the esterification reaction of short and long-chain primary alcohols (oleyl alcohol, octanol, and hexanol), for which the ester yield reduced as the length of the chain decreased. However, when they used a branched-chain alcohol (3,7-dimethyl-1-octanol) the ester yield was also reduced in comparison to primary alcohols. In our work, Eversa-mCLEA demonstrated an excellent transesterification performance using primary long-chain (octanol) and branched (isoamyl) alcohols, being a bit better when isoamyl alcohol was used. These results suggest that the specificity of Eversa for long-chain and branched alcohols could be affected by both genetic tools (by the manufacturer) and enzyme immobilization (by CLEA technique). Isoamyl alcohol was chosen for further experiments due to better performance in transesterification reactions catalyzed by Eversa-mCLEA.

### 2.4. Selection of Oil for Isoamyl Ester Synthesis

Table 3 shows the results of the transesterification reactions catalyzed by Eversa-mCLEA for the production of biolubricants using oils of different purity. It is observed that the yields in esters for both oils used in the transesterification reactions did not show significant differences in the Tukey test at 5% significance. This suggests that the changes induced by the use of the oil during cooking did not significantly affect the performance of the enzyme in the transesterification reaction, making the use of this material as a feedstock in the production of biolubricants feasible. Therefore, WCO was chosen for a further set of experiments because there are no reports in the literature on the application of this feedstock for the production of biolubricants by enzymatic transesterification. Only the production of biolubricants from WCO by two-step reactions has been reported: hydrolysis of WCO followed by esterification of the free fatty acids [1,2,73].

### 2.5. Effect of the Oil/Alcohol Molar Ratio on the Ester Yield

**Figure 2:**
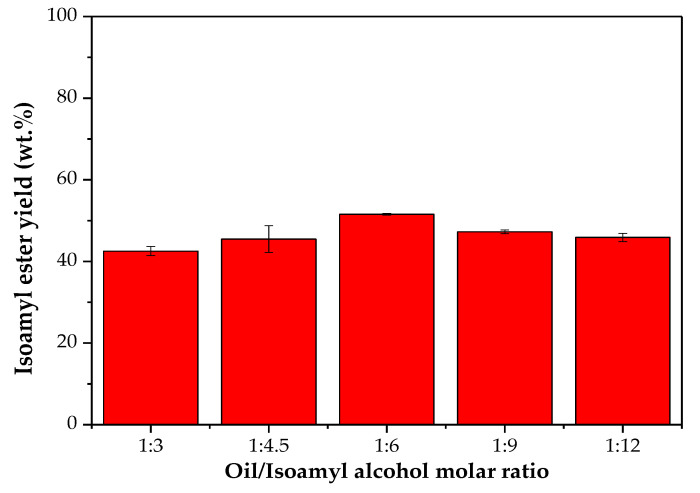
Effect of the WCO/isoamyl alcohol molar ratio in the transesterification reaction of WCO catalyzed by Eversa-mCLEA. Assay conditions: enzyme load of 2.13 U_est_/g oil, temperature of 40 °C, agitation of 250 rpm for 24 h.

The effect of different molar ratios of oil/alcohol on the isoamyl ester mass yield was also evaluated. Figure 2 shows that the highest yield of isoamyl esters (around 52 wt.%) was achieved with a molar ratio of 1:6. For the other oil/alcohol molar ratios there was a small reduction in the reaction yield. The decrease in the yields using lower amounts of alcohols may be caused by a lower saturation of the enzyme by the nucleophile, while the decrease at higher amounts of alcohol may be promoted by some negative effects of the alcohol on the enzyme structure. Thus, the WCO/isoamyl alcohol molar ratio of 1:6 was chosen for further assays.

### 2.6. Effect of Enzyme Load on the Transesterification of WCO and Isoamyl Alcohol

**Figure 3:**
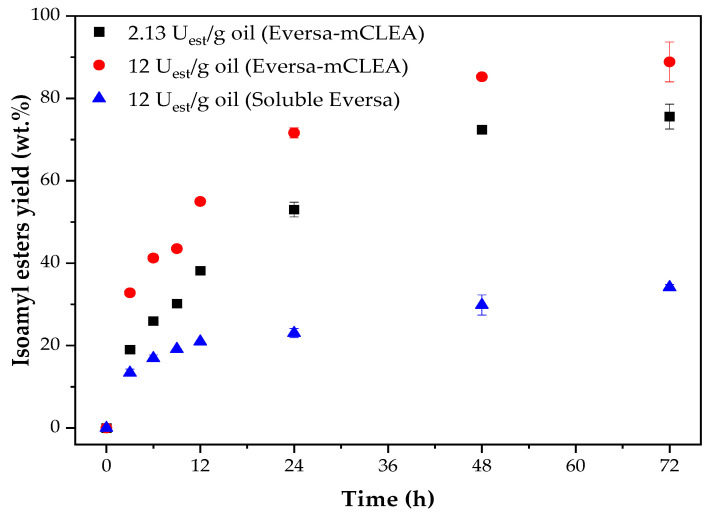
Time course of transesterification of WCO with isoamyl alcohol catalyzed by liquid Eversa and Eversa-mCLEA. Assay conditions: oil/alcohol molar ratio of 1:6, temperature of 40 °C, 1500–1750 rpm stirring in a vortex-type batch reactor.

The effect of the enzyme load on the isoamyl ester mass yield was investigated using the WCO/isoamyl alcohol molar ratio of 1:6 (Figure 3). It was observed that using Eversa-mCLEA, the higher the enzyme load was, the higher the initial transesterification rate was observed, allowing to achieve an ester yield of around 90 wt.% after 72 h of reaction.

Here, we also compared the performance of the Eversa-mCLEA with that of the liquid Eversa under the same experimental conditions. Even using the highest enzyme load evaluated, the liquid Eversa was capable of providing an ester yield of only 34.2 wt.% after 72 h of reaction. As pointed above (Section 2.2), the hydrophobic moieties on the surface of the magnetic nanoparticles could favor the transport of the hydrophobic substrates to the enzyme active site. Besides, different steric hindrances of branched alcohols for reaching the active site of liquid and immobilized Eversa should not be discarded.

Up to date, Eversa has been mainly utilized for biodiesel production using oils and fats and short-chain alcohols [26,74,75]; there is no evidence of its use in the synthesis of biolubricants [24]. This may be due to the poor performance of liquid Eversa in this reaction. However, the results of ester yields using our immobilized Eversa biocatalyst are competitive with the reported findings in the literature when using the enzymatic route for this purpose [3,76].

It should be noted that the synthesis of biolubricant using immobilized Eversa only will be viable if the mCLEA can be recycled multiple times without substantial loss of activity and this still needs to be demonstrated. Another point that still needs to be evaluated is the scale-up of the process and what devices will be adequate to efficiently separate the magnetic CLEAs at large scales. At the lab scale, the Eversa-mCLEA could be rapidly, efficiently, and easily recovered and reused (at least five times) using a neodymium magnet [26]. Aiming to scale-up the magnetic separation processes, interesting pilot-scale magnetic separators have been reported, such as “rotor-stator” high-gradient magnetic separators [77,78,79,80].

### 2.7. Characterization of the Fatty Acid Isoamyl Esters

A product prepared in this work, containing 75.60 ± 1.23 wt.% of isoamyl esters, 11.42 ± 0.61 wt.% of MAGs, 6.19 ± 0.3 wt.% of DAGs, 0.49 ± 0.14 wt.% of TAGs, and 1.73 wt.% of FFAs, was submitted to TGA/DTG and ATR-FTIR analyses.

**Figure 4:**
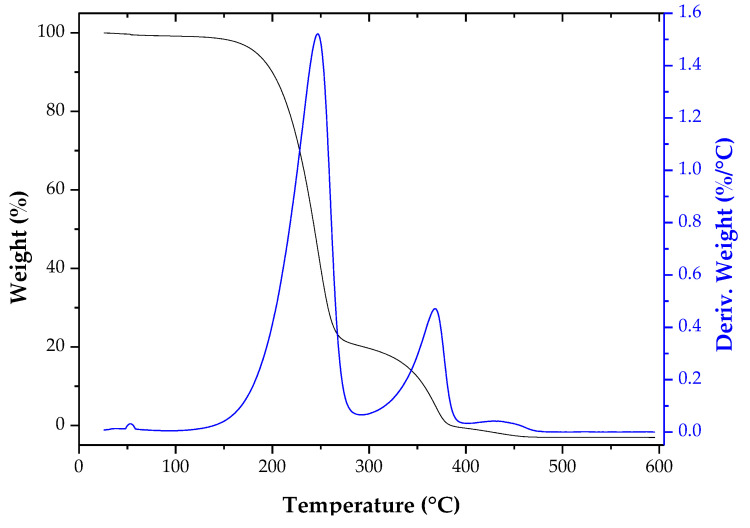
Thermogravimetric analysis for lubricant oil. Before carrying out the analysis, the material was washed with boiling water and dried at 135 °C for 16 h.

The TGA/DTG curve for the product is shown in Figure 4. The first stage of mass loss (Δm ~ 1.70%) occurred in the range of 49 °C to 53 °C, which is associated with volatile unsaponified material present in the WCO. It was observed that there were no water molecules physically/weakly adsorbed to the product. The most significant weight loss (Δm ∼ 79%) occurred in the temperature range between 115 °C to 290 °C, which is associated with the isoamyl ester molecules present in the lubricating oil. Above 300 °C, there were weight losses associated with the presence of unreacted mono-, di-, and triglycerides. Thus, the results of TGA/DTG and gas chromatography agree with each other.

**Figure 5:**
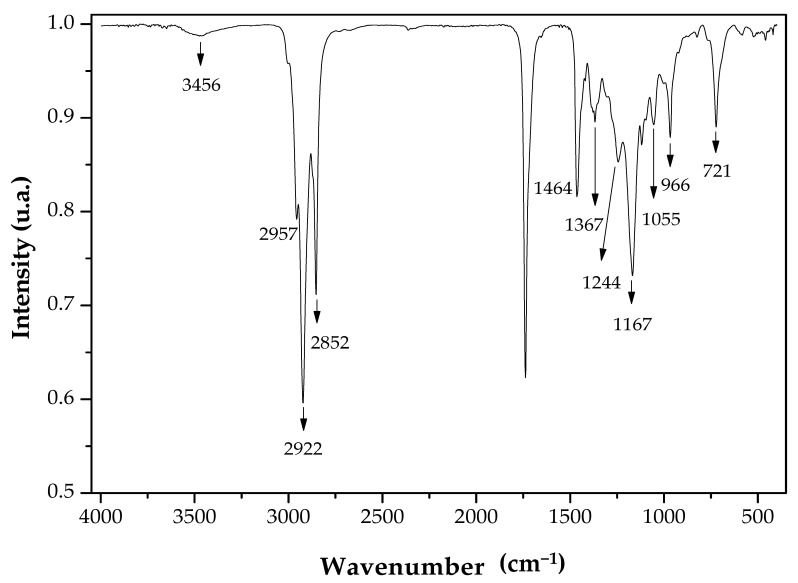
ATR-FTIR spectra of lubricant oil. Before carrying out the analysis, the material was washed with boiling water and dried at 135 °C for 16 h.

The synthesis of isoamyl esters was confirmed by ATR-FTIR spectra (Figure 5). According to the lubricating oil spectrum, it is observed that the intense band at 1738 cm^−1^ corresponds to the carbonyl group of the ester formed after the transesterification reaction, and the bands at 1244 e 1167 cm^−1^ to the stretching vibrational of –C(=O)O– group [81], thus confirming the synthesis of fatty acid isoamyl esters. In the spectrum, the band due to the O-H group present in isoamyl alcohol, which corresponds to 3456 cm^−1^ was detected at low intensity. This ATR-FTIR spectrum profile agrees with previous findings of biolubricants synthesized by esterifying oleic acid and isoamyl alcohol catalyzed by TLL [3].

## 3. Materials and Methods

### 3.1. Materials

Eversa^®^ Transform 2.0 (106.4 U_est_/g and 32.75 mg protein/mL), tert-butanol, butyric acid (99%, *w*/*w*), 1-butanol (anhydrous 99.8%, *w*/*w*), methyl heptadecanoate, Bradford reagent, PEI (average Mw ~ 25,000), and molecular sieves (rod form, size 1/16 in, 3 Å, and water adsorption capacity of 22 wt.%) were purchased from Sigma-Aldrich (St. Louis, MO, USA). Anhydrous ethanol (99.8% P.A.) and octanol were purchased from Neon (São Paulo, SP, Brazil). Methanol (P.A.), isoamyl alcohol (P.A.), and oleic acid (≥99.0% P.A.) were purchased from Synth (Diadema, SP, Brazil). Soluble starch was purchased from Qhemis (Jundiaí, SP, Brazil). Soybean oil (Liza trademark, Cargill, PR, Brazil) was acquired at the local market. WCO was provided by a pastry shop in the city of São Carlos (São Paulo, Brazil) and was vacuum filtered before being used in the experiments. Silica non-porous magnetic nanoparticles (SMNPs) functionalized with amine and octyl groups (NanoMag N(75%)-C8(25%) with a size of 30–120 nm and approximately 20 m^2^ surface area/g of support were purchased from Kopp Technologies (São Carlos, SP, Brazil). All other chemicals and solvents were of analytical grade and they were used as received.

### 3.2. Methods

All experiments were performed in duplicate. The results were expressed as an average ± standard deviation (σ). Analyses of variance between averages were performed by Tukey test at 5% significance.

#### 3.2.1. Characterization of the Oils Used in Synthesis of the Biolubricant

The oils were physically-chemically characterized in terms of acidity index [82] and saponification index [83]. Water content was determined by Karl Fisher titrimetric analysis [84] in a Titrino 907 titrator (Metrohm, Herisau, Switzerland).

#### 3.2.2. Preparation of Eversa-mCLEA

The immobilization of Eversa was carried out according to the methodology described previously [26]. A solution containing 21 mL of a PEI solution (50 mg/mL), 32 mL of liquid Eversa (32.75 ± 1.75 mg protein/mL and 127.63 ± 12.88 U_est_/mL), and 17 mL of sodium phosphate buffer (5 mM and pH 7.0) was prepared. This solution was incubated at 25 °C under stirring at 150 rpm for 60 min. Subsequently, 0.8 mL of a suspension of SMNPs (75 mg/mL), 0.128 g of starch, 1.85 mL of 5 mM sodium phosphate buffer (pH 7.0), and 1.35 mL of the enzyme solution previously treated with PEI (14.97 mg protein/mL and 58.34 U_est_/mL) were added in a Falcon tube, followed by precipitation with 12 mL of anhydrous ethanol (volumetric ratio enzyme suspension/ethanol of 1:3) in an ice bath. The suspension was stirred at 150 rpm, 4 °C in an orbital shaker (Model MA830, Marconi, Piracicaba, SP, Brazil). After 30 min, 1.51 mL of glutaraldehyde solution (50%, *v*/*v*, in water) were added and the cross-linking proceeded at 4 °C, with stirring at 150 rpm for 2.5 h. Then, the CLEAs were recovered by magnetic separation (using a neodymium magnet, 50 × 20 × 20 mm, the CLEA separation was very fast), washed once with 12 mL of sodium phosphate buffer (100 mM and pH 7.0), and resuspended in 4 mL of sodium phosphate buffer (5 mM and pH 7.0). 50 µL of α-amylase (BAN 480 L) and 50 µL of amyloglucosidase (AMG 300 L) were added to the suspension and incubated at 25 °C for 4 h to hydrolyze the starch present in the structure of the CLEAs. After, the CLEAs were recovered by magnetic separation, washed twice with 4 mL of sodium phosphate buffer (5.0 mM and pH 7.0). Then the Eversa-mCLEAs were washed twice with 4 mL of tert-butanol and kept overnight in the refrigerator for dehydration. A total of 48 tubes were prepared under the same conditions described above. The total enzyme offered to the immobilization (970.06 mg protein and 3780.43 U_est_) yielded 11.57 g of Eversa-mCLEAs with a specific activity of 106 U_est_/g biocatalyst, that is, 1226.42 U_est_, giving a recovered activity of 32.4%.

#### 3.2.3. Enzyme Activity Assays

The esterification activity was measured in terms of the synthesis of butyl butyrate following the methodology described in [85]. Free or immobilized Eversa (50 μL of liquid enzyme or 50 mg of dried CLEAs) was added to the reaction medium containing 7.5 mL of heptane, butanol (0.1 M), butyric acid (0.1 M), and molecular sieves (0.1 g). The reaction was carried out in closed glass bottles at 37 °C under 250 rpm stirring in an orbital shaker (Model MA832, Marconi, Piracicaba, SP, Brazil). After 60 min of reaction, 5 mL of ethanol were added to quench the reaction, and the acid concentration was measured by titration in a Titrino 907 titrator (Metrohm, Herisau, Switzerland) using a 20 mM KOH solution. One unit of esterification (U_est_) was defined as the initial rate of production of butyl butyrate (in μmol min^−1^) under the assay conditions.

The transesterification activities of the immobilized Eversa were assessed using the reaction between WCO and different alcohols (methanol, ethanol, octanol, and isoamyl alcohol). The reaction was carried out in closed glass bottles at 40 °C and 250 rpm stirring in an orbital shaker for 2 h. The standard transesterification conditions were oil/alcohol molar ratio of 1:6 and enzyme load of 2.13 U_est_/g oil for Eversa-mCLEA. Samples of 0.5 mL were withdrawn from the reaction mixtures every 30 min up to 2 h of reaction, and the fatty acid alkyl esters were quantified by gas chromatography. The samples were previously pretreated using phase separation, where the oil phase (esters and unreacted acylglycerols) was recovered, washed with hot distilled water (using the same volume of sample), and centrifuged (three washing steps), followed by drying overnight in an oven at 60 °C. One unit of transesterification activity (U) was defined as the initial rate of esters production (g of esters/g of sample per minute) under the conditions described previously.

#### 3.2.4. Transesterification/Esterification Reaction Using Liquid and Immobilized Eversa

The performance of liquid and immobilized Eversa was evaluated in the esterification reaction of oleic acid with octanol and isoamyl alcohol at the following conditions: enzyme load of 2.13 U_est_/g acid, oleic acid:alcohol molar ratio of 1:2, 40 °C, and stirring of 250 rpm in an orbital shaker for 24 h. In this case, molecular sieve was used to capture the water produced during esterification.

The transesterification reaction was used in order to evaluate the performance of the immobilized Eversa in the production of biolubricants using refined soybean oil and WCO as acyl donors and isoamyl alcohol. The experimental conditions were enzyme load of 2.13 U_est_/g oil, acyl donor/alcohol molar ratio of 1:6, 40 °C, and stirring of 250 rpm in an orbital shaker for 24 h. After evaluation of the different acyl donors, the effect of several WCO/alcohol molar ratios (1:3, 1:4.5, 1:6, 1:9, and 1:12) on the isoamyl ester mass yield was evaluated. In this set of experiments, an enzyme load of 2.13 U_est_/g oil was used. All reactions were carried out in closed glass bottles at 40 °C and stirring at 250 rpm. The experiments to evaluate the effect of enzyme load (2.13 and 12 U_est_/g oil) on the isoamyl ester mass yield were performed in a vortex-type batch reactor (radius ratio of 0.24, aspect ratio of 6.72) operated at 40 °C and 1500–2000 rpm stirring for 72 h. In this case, liquid Eversa was also used for comparison purpose. Samples were withdrawn to analyze glycerides (monoglycerides (MAGs), diglycerides (DAGs), and triglycerides (TAGs)), and isoamyl fatty acid esters by liquid and gas chromatography, respectively (Section 3.2.5). The FFAs contents in the biolubricant were determined according to the AOCS Official Method Ca 5a-40 [82], but modified by Rukunudin et al. [86].

#### 3.2.5. Characterization of the Biolubricant

##### Gas and Liquid Chromatography Analysis

Analyses of fatty acid esters were performed according to the ASTM D6751 and EN14103 methods [87], adapted by [26], using a 7890A Agilent chromatograph (Santa Clara, CA, USA) equipped with FID detector and Rtx-Wax capillary column (30 m × 0.25 mm × 0.25 μm, Restek Corporation, Bellefonte, PA, USA). The injector and detector were set at 250 °C. Methyl heptadecanoate was used as an internal standard. 50 mg of the washed and dried sample was dissolved in 1 mL of internal standard solution (10 mg mL^−1^, in heptane) and 1 μL was injected into the equipment. The ester mass yield (wt.%) was calculated according to Equation (1):

(1) $$\mathrm{Ester}\;\mathrm{yield}\;(\%)\;=\;\frac{\left(\sum A\right){\;-\;A}_{\mathrm{SI}}}{A_{\mathrm{SI}}}\times \frac{C_{\mathrm{SI}\;}{\times \;V}_{\mathrm{SI}}}{m}\times \;100\;$$

where ΣA is the total peak area of fatty acid esters C14:0 to C24:0, A_SI_ is the peak area of the internal standard (methyl heptadecanoate, C17), C_SI_ is the concentration of the internal standard (10 mg·mL^−1^), V_SI_ is the volume of the internal standard solution (1 mL), and m is the mass of sample (50 mg).

The analysis and quantification for samples containing TAGs, DAGs, and MAGs (in wt.%) were performed according to the methods described by Holcapek et al. [88]. The E-2695 Waters chromatograph (Waters, Millford, CA, USA) equipped with a UV detector (set to 205 nm) and Ascentis^®^Express C18 reverse-phase column (10 cm × 46 mm × 2.7 μm, Sigma-Aldrich, St. Louis, MO, USA) was used in the analysis. The mobile phase was composed of water (Phase A), acetonitrile (Phase B), and isopropanol:hexane (5:4, *v*/*v*) (Phase C) with a flow rate of 1 mL·min^−1^. 50 mg of the washed and dried sample was diluted 1650 times in a 2-propanol-hexane solution (5:4, *v*/*v*) and 20 μL was injected into the equipment.

##### ATR-FTIR and TGA Analyses

In order to check the transesterification reaction, a Fourier Transform Infrared spectrophotometer (ATR-FTIR) (model Vertex 70, Bruker, Germany) with a diamond crystal single bounce accessory was used. The samples were directly applied to the crystal cell. ATR-FTIR spectra for isoamyl ester were acquired after 32 scans between 4000 and 400 cm^−1^ with a spectral resolution of 4 cm^−1^. Thermal analyses were carried out using a TGA Q500 thermogravimetric analyzer (TA Instruments) in a nitrogen atmosphere from room temperature up to 600 °C using a heating rate of 10 °C·min^−1^.

## 4. Conclusions

Eversa is an enzyme launched by the biochemical industry to produce biodiesel from acid oily feedstocks and short-chain alcohols (mainly methanol). However, using longer alcohols, the free enzyme showed the worst performance in esterification and transesterification reactions than the Eversa after immobilization via CLEAs technique. Now, the enzyme performance in this reaction becomes very suitable for applied purposes.

Thus, this paper shows the feasibility of the enzymatic approach used for the production of biolubricants from the transesterification of the WCO and isoamyl alcohol when utilizing Eversa-mCLEA, which becomes around three-fold more active in this reaction than the free enzyme. The synthesis of fatty acid isoamyl esters was successfully demonstrated using immobilized Eversa (around 90 wt.% of ester yield), while the liquid Eversa had a poorer performance (around 34 wt.% of ester yield). The nature of the product was confirmed by spectroscopy (ATR-FTIR) and chromatography (GC) analyses. Furthermore, our findings make the use of fusel oil (a by-product of the distillation of ethanol composed mostly of isoamyl alcohol [89,90]) attractive as an acyl acceptor in the synthesis of biolubricants [64].

## Figures and Tables

**Table 1:** Physicochemical analysis for oleic acid, refined soybean oil, and waste cooking oil.

Parameter	Oleic Acid	Refined Oil	Waste Cooking Oil
Acidity index (mgKOH/g)	≥99.0 ^a^	0.12 ± 0.0048	3.93 ± 0.08
Saponification index (mgKOH/g)	206.53 ± 4.97	195.31 ± 1.53	189.77 ± 8.4
Water content (%)	0.15 ± 0.0011	0.056 ± 0.0029	0.081 ± 0.0034

^a^ Source: Synth (Diadema, SP, Brazil).

**Table 2:** Esterification of oleic acid catalyzed by liquid Eversa and Eversa-mCLEA using octanol and isoamyl alcohol as acyl acceptors. Assay conditions: enzyme load of 2.13 U_est_/g oil, acid/alcohol molar ratio of 1:2, temperature of 40 °C, agitation of 250 rpm for 24 h, and molecular sieve as a desiccant.

Alcohol	Ester Yield (wt.%)	
	Liquid Eversa	Eversa-mCLEA
Octanol	34.97 ± 0.96	51.18 ± 1.18
Isoamyl	36.54 ± 0.89	52.71 ± 0.72

**Table 3:** Selection of oils for the transesterification reaction catalyzed by Eversa-mCLEA using isoamyl alcohol as acyl acceptor. Assay conditions: enzyme load of 2.13 U_est_/g oil, oil/isoamyl alcohol molar ratio of 1:6, temperature of 40 °C, agitation of 250 rpm for 24 h.

Acyl Donors	Ester Yield (wt.%) *
Refined soybean oil	52.60 ^a^
Waste cooking oil	51.55 ^a^

* The index ^a^ means that are no statistical differences in the average values using the Tukey test (5% significance).

## Data Availability

The data presented in this study are available on request from the corresponding author.

## Sample Availability

Samples of the compounds are not available from the authors.
